# Stress and Sleep Deprivation Experienced by Bus Drivers of Government Bus Depots in an Urban Area of Chengalpattu District in South India

**DOI:** 10.7759/cureus.67689

**Published:** 2024-08-24

**Authors:** Bharathwaj P, Pradeep M. V. M., Kaveri P, Anantharaman V. V., Logaraj M

**Affiliations:** 1 Community Medicine, SRM Medical College Hospital and Research Centre, SRM Institute of Science and Technology, Chengalpattu, IND

**Keywords:** epworth sleepiness scale, perceived stress scale, sleep deprivation, perceived stress, bus drivers

## Abstract

Background

Driving a bus is far more stressful than other professions. Bus drivers also take more sick leave than other workers because of the physical and mental demands of their employment. Bus drivers are especially vulnerable because of their intense work environment, poor sleep, and poor food pattern. The aim of the study was to assess stress and sleep deprivation of the government bus drivers in Chengalpattu District in Tamil Nadu and to identify the sociodemographic factors influencing them.

Methodology

An analytical cross-sectional study was conducted among 429 government bus drivers working under Tamil Nadu State Transport Corporation (TNSTC )in selected government bus depots in the urban area of Chengalpattu District in Tamil Nadu, India. The principal investigator collected the data by using a semi-structured questionnaire which comprises six parts, i.e., sociodemographic variables, job-related factors, comorbidity, habits, stress assessed by using the Perceived Stress Scale (PSS-10), and sleep deprivation assessed by using the Epworth Sleepiness Scale (ESS).

Results

In the present study, the mean age of participants was 47.683 years. Hindus comprise 95.3% of the sample, and 96.7% are married. Approximately 47.6% of the drivers have achieved a higher secondary education. The average time spent working as a heavy vehicle driver was 20.4 years. About 37.1% (159 individuals) have hypertension, making it the most prevalent comorbidity. In this study, 47.3% (203 participants) reported very poor sleep, 35.7% (153 participants) reported average sleep, and 17.0% (73 participants) reported good sleep. Inferential statistics revealed that those drivers who were degree holders took less than three breaks in duty time, slept over six hours at night, had the habit of smoking and drinking alcohol, and took more than 60 minutes to fall asleep experienced very poor sleep according to the ESS. In this study, 57.1% (245 participants) reported moderate stress levels, 24.2% (104 participants) showed high levels of perceived stress, and 18.6% (80 participants) reported low stress levels. Inferential statistics revealed that those drivers who were Muslims, degree holders, those with primary education, smokers, alcohol consumers, drivers with very poor sleep, and those who took more than six days of casual leave in the past six months experienced high stress according to the PSS-10.

Conclusion

Implementing effective health management strategies and minimizing work-related stress will lead to a decrease in work-related disorders among drivers. TNSTC should ensure enough lodging facilities for drivers at depots, bus terminals, and outstations. They require a minimum of eight hours of sleep every day to maintain optimal physical well-being.

## Introduction

Compared to many other professions, bus driving is more demanding. This demanding job requires a high level of skill and constant vigilance to respond to the immediate environment, including frequent braking, accelerating, and maneuvering through crowded areas. Ensuring passenger safety adds significant mental and physical strain throughout the day. Additional stressors include uncomfortable seating, varying weather, strict adherence to schedules, disputes with passengers, and conflicts between drivers and conductors. These factors collectively contribute to the high levels of psychological stress experienced by bus drivers [1].

Bus drivers frequently work double or extra shifts. This can be driven by financial pressures from their families or the need to cover for absent colleagues. Such extensive work commitments negatively affect their relationships with peers and family members, leading to social isolation and reduced well-being [2]. A range of health issues are closely linked to the high job strain and psychological stress bus drivers endure. Among the most common health problems they face are chronic respiratory illnesses, gastrointestinal disorders, hypertension, type 2 diabetes, and cardiovascular diseases. The demanding nature of their job and the associated stress often exacerbate these health challenges [3].

Additionally, the physical and mental toll of their work leads bus drivers to take sick leave more frequently than workers in other professions. The combination of long hours, high stress, and health complications creates a vicious cycle that can severely affect their overall quality of life. This frequent need for time off further strains their professional relationships and contributes to a precarious balance between their work and personal lives [4].

Beyond the inherently stressful working conditions, bus drivers also suffer from poor sleep and unhealthy eating habits, rendering them a vulnerable group. In India, approximately 80% of bus drivers experience varying levels of psychological stress. This stress often manifests in dangerous behaviors such as racing, speeding, and difficulty reacting appropriately to their immediate surroundings, which significantly increases the risk of road traffic accidents and jeopardizes the safety of all passengers [5].

Chronic psychological stress directly affects sleep patterns, creating a feedback loop where poor sleep further exacerbates stress. In India, around 62% of bus drivers experience daytime sleepiness while on duty, and about 15% struggle to fall asleep at night. As a coping mechanism, roughly 19% of these drivers resort to smoking or chewing tobacco [6].

Persistent stress and a lack of leisure time with family and friends can lead to depression. This combination of insufficient sleep, high stress, and depression not only affects their personal health but also contributes to an increased number of road traffic accidents, a growing public health concern in India [7,8]. Against the backdrop outlined above, a study was undertaken to quantitatively assess the levels of stress and sleep deprivation experienced by government bus drivers in Chengalpattu District, Tamil Nadu, and to analyze the influence of various sociodemographic factors on these conditions, using validated scales such as the Perceived Stress Scale (PSS-10) and Epworth Sleepiness Scale (ESS).

## Materials and methods

Study settings and duration

An analytical cross-sectional study was carried out among bus drivers employed by the Tamil Nadu State Transport Corporation (TNSTC) in the Kilambakkam and Tambaram government bus depots in the urban area of Chengalpattu District in Tamil Nadu during the period from March 2023 to March 2024.

Sample size calculation

Joel et al. conducted a cross-sectional investigation on a group of 100 urban bus drivers [9]. The results of the PSS-10 show that 49% experienced severe levels of stress. We determined the sample size based on the prevalence rate given above by using the formula N = 3.84 * p * q / d2, where p is the prevalence rate, q is the complement of p, and d is the accuracy (with a 5% absolute error). To account for the nonresponse rate, a 10% increase was applied to the sample, resulting in a minimum requirement of 423 samples for the study. A total of 429 samples of government bus drivers in selected government bus depots were included in the present study.

Inclusion criteria

We included government bus drivers, those who are working under TNSTC in Chengalpattu District having work experience for more than one year, and those who were willing to participate by giving consent.

Exclusion criteria

We excluded those bus drivers who were not willing to take part in the study or give consent and those who were not available even after three visits.

Sampling method

The convenient sampling was used to select the samples in selected government bus depots in the urban area of Chengalpattu District in Tamil Nadu. Only two government bus depots (Kilambakkam and Tambaram bus depots) out of four in Chengalpattu District were included as per permission obtained. These depots were selected because they were closer to our institution and had drivers available in high number. Bus drivers were approached on-site, and those who met the inclusion criteria and consented to participate were included in the study

Data collection procedure

Data collection was conducted through face-to-face interviews by the principal investigator. Each interview lasted approximately for 35 minutes and was conducted in a private setting to ensure confidentiality. The semi-structured questionnaire, consisting of six sections, was administered consistently across all participants to minimize interviewer bias. These sections include sociodemographic variables, job-related factors, comorbidity, habits, stress assessed using the PSS-10 and sleep deprivation assessed using the ESS. The questionnaire was pretested on a sample of 40 bus drivers similar to the study population to identify any issues with comprehension or the wording of questions. The questionnaire was appropriately revised as needed, and the interview was performed according to the final revised schedule.

Study tools

Part 1: Sociodemographic Variables

These variables include completed age, gender, marital status, place of residence, education, and religion.

Part 2: Job-Related Factors

These factors include years of experience, distance travelled per day, duration of shift working, number of days of casual leaves, and number of breaks in duty time and refreshments in between driving.

Part 3: Comorbidities

This part includes hypertension, bronchial asthma, diabetes mellitus, cardiovascular disease, and other morbidity.

Part 4: Habits

The habits comprise smoking and consumption of alcohol.

Part 5: PSS-10

The PSS-10 has been extensively utilized and psychometrically verified as a dependable assessment of psychological stress experienced within the past four weeks [10]. The survey comprises 10 areas that are assessed using a five-point Likert scale, with 0 representing "never" and 4 representing “very often.” The PSS-10 construct exhibits a dual-component structure, with the first factor being "general stressors" and the second factor being "the ability to cope" [11].

The PSS-10 score is obtained by summing the scores of all the items, with reverse coding for items 4, 5, 7, and 8, as they are positively stated. The PSS-10 score ranges from zero to 40, with the 40-point score representing the highest perceived stress level. The PSS-10 does not have any diagnostic cutoff to differentiate between the stressed and not stressed individuals [12].

Part 6: ESS

The ESS is a self-administered survey that assesses daytime drowsiness by prompting participants to indicate their likelihood of dozing off during eight typical everyday activities. The questionnaire utilizes a four-point Likert response format, with response options ranging from zero to three. The total score achievable on the assessment is 24. A score of 11 or above on the ESS shows the presence of excessive daytime sleepiness [13].

Ethical consideration

Data collection was conducted after obtaining consent from the scientific and ethical committee of the SRM Medical College Hospital and Research Centre (clearance number: SRMIEC-ST0323-502). Informed consent was obtained from all participants after they were provided with detailed information about the study's purpose, procedures, potential risks, and benefits. Participants were assured of the confidentiality of their responses and their right to withdraw from the study at any point without any repercussions.

Data analysis

All data were entered in MS Excel (Microsoft Corporation, Redmond, Washington, United States) and analyzed in IBM SPSS Statistics for Windows, Version 26 (Released 2019; IBM Corp., Armonk, New York, United States). Categorical variables were represented using frequency and proportion, whereas continuous variables were represented using mean and standard deviation. In inferential statistics, the data was analyzed to determine if there were statistically significant differences between discrete variables in the two groups. This was done using either the Chi-Square test or Fisher's exact test. All statistical procedures used a significance level of 0.05 for the probability value.

## Results

The mean age of the 429 study participants was 47.683 years, with a standard deviation of 21.91. A majority of the participants are Hindu, comprising 95.3% of the sample (409), and 415 participants, or 96.7%, were married. Participants who have completed secondary education comprise 39.6% (170 participants) of the total, while those who have completed higher secondary education comprise 47.6% (204 participants). Degree holders comprise 7.2% of the sample (31 participants), while individuals with only primary education comprise 5.6% (24 participants). In the present study, 57.1% of the participants (n = 245) were smokers, and 38.5% of the participants (n = 165) were alcoholics. Hypertension was the most prevalent comorbidity among the bus drivers, with 37.1% (159 individuals) exhibiting it. Cardiovascular disease was present in 20.5% (88 participants),, while diabetes mellitus affected 28.5% (122 participants). A total of 27.5% of the participants (118) reported that they did not have any comorbidities. Table 1 illustrates the sociodemographic distribution of the study participants.

**Table 1:** Sociodemographic distribution of the study participants (n = 429)

Variables		Frequency	Percent
Age in years (mean ± SD)		47.68 ± 21.9	
Religion	Hindu	409	95.3
	Christian	12	2.8
	Muslim	8	1.9
Marital status	Married	415	96.7
	Unmarried	7	1.6
	Divorced	7	1.6
Education	Degree holder	31	7.2
	Higher secondary	204	47.6
	Secondary	170	39.6
	Primary	24	5.6
Smoking	Yes	245	57.1
	No	184	42.9
Consumption of alcohol	Yes	165	38.5
	No	264	61.5
Comorbidities	Bronchial asthma	22	5.3
	Cardiovascular disease	88	20.5
	Diabetes mellitus	122	28.5
	Hypertension	159	37.1
	No	118	27.5

The mean number of years of experience as heavy vehicle drivers was 20.49 years. The average daily distance traveled was 335.5 kilometers. The distribution of the study participants based on the casual leave taken in the past three months indicates that 48.7% (209 participants) took less than three days of casual leave and 38.5% (165) took between three and six days of absence. Only 12.8% (55 participants) utilized casual leave for a period exceeding six days. The number of breaks taken during duty time is as follows: 80.0% (343 participants) take fewer than three breaks, while 19.3% (83 participants) take between three and five breaks. The number of participants who take more than five breaks is extremely low, at 0.7% (three participants). 92.5% of the participants (397) consume tea or coffee as a refreshment between driving sessions. Only 0.5% (two participants each) explicitly engage in smoking, while 6.5% (28 participants) engage in pan chewing. The majority of the samples (50.8%) slept for less than four hours per night, with 218 individuals falling into this category. Furthermore, 151 participants (35.2%) reported sleeping for four to six hours each night. Only 60 participants (14.0%) slept for more than six hours each night. Table 2 shows the job-related factors of bus drivers.

**Table 2:** Job-related factors of bus drivers (n = 429) *Mean and standard deviation

Variables		Frequency	Percent
Years of experience		20.49 ± 7.22*	
Total distance traveled everyday		335.5 ± 136.42*	
Number of casual leaves taken in the past 3 months	3 to 6 days	165	38.5
	Less than 3 days	209	48.7
	More than 6 days	55	12.8
Number of breaks taken in duty time	3 to 5	83	19.3
	Less than 3	343	80
	More than 5	3	0.7
Refreshment between driving sessions	Pan chewing	28	6.5
	Smoking	2	0.5
	Tea/coffee	399	93
Duration of sleep at night	4 to 6 hours	151	35.2
	less than 4 hours	218	50.8
	more than 6 hours	60	14

Approximately 47.3% of the participants (203 in total) reported experiencing extremely poor sleep, which implied the necessity of medical help. In addition, 35.7% (153 participants) reported an average sleep quality, while 17.0% (73 participants) described their sleep quality as good. About 57.1% (245 bus drivers) experience moderate levels of stress according to the PSS-10. In addition, 24.2% of the participants (104 individuals) experienced high levels of perceived stress, whereas 18.6% (80 individuals) reported low levels of stress. Table 3 displays the distribution of the study participants based on the categories of the ESS and PSS-10.

**Table 3:** Distribution of the study participants according to the Epworth Sleepiness Scale category and Perceived Stress Scale category (n = 429)

Outcome variables	Frequency	Percent
Epworth Sleepiness Scale category		
Average sleep	153	35.7
Good sleep	73	17
Very poor sleep	203	47.3
Perceived Stress Scale category		
High stress	104	24.2
Low stress	80	18.6
Moderate stress	245	57.1

Table 4 shows the association between various variables and the ESS categories. Among religions, Hindus form the majority, with Muslims showing the highest percentage (75%) of very poor sleep. This difference in the proportion of sleep patterns between the religions was not statistically significant (p-value = 0.477). Among marital status, married form the majority (96.7%), with divorced people showing the highest percentage (57.1%) of very poor sleep. This difference in the proportion of sleep patterns between marital statuses was not statistically significant (p-value = 0.707). Education shows a significant variation, with degree holders having the highest percentage (61.3%) of poor sleep. This difference in the proportion of sleep patterns between the educational statuses was statistically significant (p-value = 0.029). Less than three days of casual leave in the past three months has shown the highest percentage (52.2%) of very poor sleep. This difference in the proportion of sleep pattern between the number of casual leaves taken in the past three months was not statistically significant (p-value = 0.33). Less than three breaks in working time show the highest percentage (49.3%) of very poor sleep. This difference in the proportion of sleep patterns between the number of casual leaves taken in the past three months was statistically significant (p-value = 0.008). Refreshment habits, like tea and coffee consumption, are common (47.6%) among the drivers. This difference in the proportion of sleep patterns between the number of refreshment habits was not statistically significant (p-value = 0.138). Night sleep duration is crucial, but those sleeping more than six hours at night show very poor sleep at work (70%). The difference in the proportion of sleep patterns between the duration of sleep at night was statistically significant (p-value = 0.0001). Cigarette smoking shows a strong association, with higher consumption correlating with poorer sleep (54.3%). This difference in the proportion of sleep pattern between the number of cigarettes smoked per day was statistically significant (p-value = 0.001). Alcohol consumption is significantly associated with drinkers being more likely to have very poor sleep (57.6%). This difference in the proportion of sleep patterns between the habit of drinking alcohol was statistically significant (p-value = 0.007).

**Table 4:** Association between various sociodemographic variables and the Epworth Sleepiness Scale categories *Fisher exact test was used. ^#^Statistically significant at p-value <0.05

Epworth Sleepiness Scale category vs sociodemographic determinants			Epworth Sleepiness Scale category			Total, n = 429	Chi-square value	p-value
			Average sleep (n = 153)	Good sleep (n = 73)	Very poor sleep (n = 203)			
Religion	Christian	Count	5	3	4	12	5.329*	0.477
		%	36.40%	27.30%	36.40%	100%		
	Hindu	Count	146	70	193	409		
		%	35.70%	17.10%	47.20%	100%		
	Muslim	Count	2	0	6	8		
		%	25.00%	0.00%	75.00%	100%		
Marital status	Divorced	Count	1	2	4	7	2.133*	0.707
		%	14.30%	28.60%	57.10%	100%		
	Married	Count	149	70	196	415		
		%	35.90%	16.90%	47.20%	100%		
	Unmarried	Count	3	1	3	7		
		%	42.90%	14.30%	42.90%	100%		
Education	Degree holder	Count	3	9	19	31	14.021*	0.029^#^
		%	9.70%	29.00%	61.30%	100%		
	Higher secondary	Count	72	30	102	204		
		%	35.30%	14.70%	50.00%	100%		
	Primary	Count	9	6	9	24		
		%	37.50%	25.00%	37.50%	100%		
	Secondary	Count	69	28	73	170		
		%	40.60%	16.50%	42.90%	100%		
Number of casual leaves taken in the past 3 months	3 to 6 days	Count	65	33	67	162	6.518	0.33
		%	39.50%	19.80%	40.70%	100%		
	Less than 3 days	Count	70	30	109	209		
		%	33.50%	14.40%	52.20%	100%		
	More than 6 days	Count	18	10	27	55		
		%	32.70%	18.20%	49.10%	100%		
Number of breaks taken in duty time	3 to 5	Count	27	23	33	81	14.030^*^	0.008^#^
		%	33.30%	27.20%	39.50%	100%		
	Less than 3	Count	126	48	169	343		
		%	36.70%	14.00%	49.30%	100%		
	More than 5	Count	0	2	1	3		
		%	0.00%	66.70%	33.30%	100%		
Refreshment between driving sessions	Pan chewing	Count	11	5	12	28	6.943*	0.138
		%	39.30%	17.90%	42.90%	100%		
	Smoking	Count	1	0	1	2		
		%	50.00%	0.00%	50.00%	100%		
	Tea/coffee	Count	141	68	190	399		
		%	35.30%	17.00%	47.70%	100%		
Duration of sleep at night	4 to 6 hours	Count	66	31	54	151	29.340^*^	0.0001^#^
		%	43.30%	20.70%	36.00%	100%		
	Less than 4 hours	Count	80	31	107	218		
		%	36.70%	14.20%	49.10%	100%		
	More than 6 hours	Count	7	11	42	60		
		%	11.70%	18.30%	70.00%	100%		
Habit of smoking	No	Count	70	44	70	184	15.375	0.001^#^
		%	40.80%	18.50%	40.80%	100%		
	Yes	Count	83	29	133	245		
		%	33.90%	11.80%	54.30%	100%		
Habit of drinking alcohol	No	Count	108	48	108	264	12.454	0.007^#^
		%	40.80%	18.50%	40.80%	100%		
	Yes	Count	45	25	95	165		
		%	27.30%	15.20%	57.60%	100%		

Table 5 shows the association between various sociodemographic variables and PSS-10 categories. Religion showed a notable association (p-value 0.043), with Muslims reporting higher proportions of high stress (75%) compared to Hindus and Christians. Divorced people had higher stress (28.6%) than other unmarried and married people. This difference in the proportion of PSS-10 between marital status was statistically not significant (p-value = 0.876). Education level also showed an association (p-value 0.084), showing that degree holders had high stress levels (35.5%). The number of casual leaves taken in the past three months (p-value = 0.018) showed significant associations with perceived stress levels. Casual leaves taken more than six days in the past three months had higher stress levels (36.4%). Longer duration of sleep at night (p-value = 0.0001) showed significant associations with perceived stress levels. Longer duration of sleep at night had higher stress levels (63.3%). Smoking habits (p-value = 0.001) showed significant associations with perceived stress levels. Smoking habits had higher stress levels (50%) and showed significant associations with perceived stress levels. The habit of drinking alcohol had higher stress levels (33.3%) and showed significant associations (p-value = 0.006) with perceived stress levels. The ESS category (p-value = 0.0001) showed significant associations with perceived stress levels. Very poor sleep had higher stress levels (42.4%). 

**Table 5:** Association between various sociodemographic variables and Perceived Stress Scale categories ^*^Statistically significant at p-value <0.05

Perceived stress scale category vs sociodemographic determinants			Perceived stress scale category			Total	Fisher’s exact value	p-value
			High stress (n = 104)	Low stress	Moderate stress	(n = 429)		
				-80	(n = 245)			
Religion	Christian	Count	3	3	6	12	10.823	0.043*
		%	27.30%	27.30%	45.50%	100%		
	Hindu	Count	95	77	237	409		
		%	23.20%	18.80%	57.90%	100%		
	Muslim	Count	6	0	2	8		
		%	75.00%	0.00%	25.00%	100%		
Marital status	Divorced	Count	2	2	3	7	1.553	0.876
		%	28.60%	28.60%	42.90%	100%		
	Married	Count	101	77	237	415		
		%	24.30%	18.60%	57.10%	100%		
	Unmarried	Count	1	1	5	7		
		%	14.30%	14.30%	71.40%	100%		
Education	Degree holder	Count	11	9	11	31	10.701	0.084
		%	35.50%	29.00%	35.50%	100%		
	Higher secondary	Count	52	30	122	204		
		%	25.50%	14.70%	59.80%	100%		
	Primary	Count	4	7	13	24		
		%	16.70%	29.20%	54.20%	100%		
	Secondary	Count	37	34	99	170		
		%	21.80%	20.00%	58.20%	100%		
Number of casual leaves taken in past 3 months	3 to 6 days	Count	27	34	104	165	11.956	0.018*
		%	16.40%	20.60%	63.00%	100%		
	less than 3 days	Count	57	35	117	209		
		%	27.30%	16.70%	56.00%	100%		
	more than 6 days	Count	20	11	24	55		
		%	36.40%	20.00%	43.60%	100%		
Duration of sleep at night (in hours)	4 to 6 hours	Count	26	32	93	151	61.769	0.0001*
		%	17.20%	21.20%	61.60%	100%		
	Less than 4 hours	Count	40	38	140	218		
		%	18.30%	17.40%	64.20%	100%		
	More than 6 hours	Count	38	10	12	60		
		%	63.30%	16.70%	20.00%	100%		
Habit of smoking	No	Count	31	46	107	184	14.29	0.001*
		%	16.90%	25%	58.10%	100%		
	Yes	Count	73	34	138	245		
		%	29.80%	13.90%	56.30%	100%		
Habit of drinking alcohol	No	Count	49	55	160	264	12.91	0.006*
		%	18.50%	21.20%	60.40%	100%		
	Yes	Count	55	25	85	165		
		%	33.30%	15.20%	51.50%	100%		
Epworth sleepiness scale category	Average sleep	Count	14	17	122	153	176.585	0.0001*
		%	9.20%	11.10%	79.70%	100%		
	Good sleep	Count	4	47	22	73		
		%	5.50%	64.40%	30.10%	100%		
	Very poor sleep	Count	86	16	101	203		
		%	42.40%	7.90%	49.80%	100%		

## Discussion

Comparison of sleep deprivation among drivers with similar studies

According to this study, 47.3% (203 participants) are classified as having a very low quality of sleep. This data emphasizes a substantial proportion of the study group facing sleep-related problems that need to be addressed, which might affect their general health and well-being. A self-administered questionnaire study was conducted on a sample of 4331 drivers employed in the commercial bus and truck industry. According to the ESS, more than 45% of drivers were categorized as experiencing excessive daytime sleepiness, with an ESS score of more than 11. Individuals with an ESS score greater than 11 exhibited a substantial correlation with experiencing drowsiness, falling asleep while driving, and being involved in accidents. The primary factor contributing to tiredness, as indicated by the study conducted by Leechawengwongs et al., was sleep loss, accounting for 90% of cases [14].

Vennelle et al. conducted a survey with 677 bus drivers. They noted that 8% of drivers experienced drowsiness and fell asleep while driving at least once a month, while 7% had been involved in an accident, and 18% had narrowly avoided an accident because of tiredness while working. Bus drivers exhibited a significant prevalence of tiredness and sleep-related accidents, as determined by the researchers [15].

According to inferential statistics, drivers who had a degree, took less than three breaks during their duty time, slept for more than six hours at night, wore glasses while driving, had a habit of smoking cigarettes and drinking alcohol, experienced very poor sleep according to the ESS, and require medical attention.

Mani et al. conducted a study on bus drivers in Malaysia in which the ES was utilized to quantify the level of sleepiness. A total of 85 participants were included in the study, and out of these, 18 individuals (21.2%) were identified as experiencing sleepiness. The drowsiness level among express bus drivers was influenced by characteristics such as body mass index, sleep amount, and job experience, as indicated by the result [16].

Miller et al. conducted a study that investigated the frequency and origins of weariness among bus drivers in London [17]. A total of 20.8% of the participants reported experiencing drowsiness on a frequency of at least 2-3 times per week. There were other reasons that could have contributed to tiredness, including work-related factors, sleep-related factors, and personal issues. Some specific personal factors included not getting enough rest (less than 11 hours) between shifts, working for six or more consecutive days without a day off, and reporting poor health. Their conclusion was that bus driver weariness is a complex and prevalent issue that has to be tackled.

Bus drivers are a significant subgroup of workers who work in shifts, and if they have an undetected sleep condition, it could cause road accidents. Studies have shown that even minor tiredness can impair performance to the same extent as alcohol intoxication [18].

TNSTC can provide enough driver lodging at depots, bus terminals, and outstations. Rising smartphone games and browsing use by middle-aged drivers during free time and after work is adding to sleep deprivation. Drivers need eight hours of sleep per day to stay healthy. Sleep debt makes drivers tired and drowsy, increasing accident risk. It also causes tension, which impairs driving [19].

Thus, this study is the first step in identifying undiagnosed work-related sleep disturbances among public transport drivers. The simple questionnaire-based procedures in this study could be used for preemployment and periodic screening of night shift drivers to ensure quick referral for sleep disorder assessment and treatment. Drowsiness-related road accidents would drop dramatically with this intervention. A database of driver sleepiness patterns and frequency could help adapt work schedules to avert accidents and close calls.

Comparison of perceived stress among drivers with similar studies

The study participants were categorized based on their PSS-10 ratings, revealing a mean score of 19.918. These findings show that, on average, participants in the study reported experiencing moderate levels of perceived stress. However, there was considerable heterogeneity in stress levels across individuals within the study sample. Joshi and Vaidya conducted a cross-sectional study with 260 bus drivers in Pune, India [20]. The average score of perceived stress among bus drivers was 25.23. The data clearly indicates that bus drivers had a significantly high PSS-10 score.

About 24.2% (104 participants) experienced high levels of perceived stress in this study. This data emphasizes the frequency of different levels of stress among the participants in the study, emphasizing the important influence of felt stress on the mental and physical health of individuals. Joel et al. conducted a cross-sectional investigation on the group of 100 urban bus drivers [9]. The results of the PSS-10 indicate that 49% experienced severe levels of stress. The study also shows a substantial correlation between stress levels and low sleep quality. Joshi et al. conducted a cross-sectional study in India with a sample of 140 auto-rickshaw drivers selected at random. According to Cohen’s Perceived Stress Score, 18 individuals (12.9%) experienced a high level of stress. Furthermore, they observed that there was no noteworthy correlation between any sociodemographic characteristics and the PSS-10 score [21]. The study conducted by Premnath and Rajkumar found that a significant majority (65.88%) of the employed individuals experience high levels of stress [22].

Based on inferential statistics, it was found that drivers who identified as Muslims, held a degree, had smoked, consumed alcohol, had very poor sleep, and took more than six days of casual leaves in the past six months experienced high levels of stress according to the PSS-10. These drivers require medical attention. The results indicate that the way participants perceive stress is affected by a mix of sociodemographic, behavioral, and health-related factors. This emphasizes the intricate relationship in determining stress levels in this particular group.

Sabarinathan et al. conducted a survey including 150 employees in the transportation sector. The results indicate job stress-related features had minimal influence on demographic variables. However, age, marital status, and monthly wage had a significant impact on the job satisfaction of bus drivers. It was determined that all bus drivers feel occupational stress, irrespective of demographic considerations [23]. The Lazarus transactional model of psychological stress posits that stress arises when an individual's perception of the demands of a task surpasses their ability to cope with the situation. People's answers vary based on their ability to adapt to the situation. The level of stress experienced by a driver is influenced by the interplay between situational and personal factors [24].

The stress experienced by drivers is caused by a demanding route schedule, inadequate opportunities for brief breaks, traffic congestion, and a lack of time to attend to their food and restroom needs. Implementing effective health management strategies and minimizing work-related stress will lead to a decrease in the occurrence of work-related disorders among drivers. Prolonged stress can heighten the likelihood of promoting cancer cell growth within the body [25]. The personnel should take part in beneficial training sessions and workshops to decrease their stress levels. To mitigate their stress levels, employees must comprehend the nature of their profession and its societal significance. If their stress level decreases, production will increase.

Strength

One of the key strengths of this study is the large sample size of 429 bus drivers, which provides a robust dataset and enhances the generalizability of the findings. Additionally, the use of validated instruments such as the PSS-10 and the ESS adds credibility to the assessment of stress and sleep deprivation among the participants. The study also addresses an important public health issue by focusing on a high-risk occupational group, offering insights that can inform targeted interventions.

Limitation

Despite its strengths, this study has several limitations that should be considered. The use of convenience sampling may limit the generalizability of the findings to other populations or regions. Recall bias is another potential limitation, as participants may have inaccurately reported their stress levels or sleep patterns, either due to forgetfulness or a desire to present themselves in a more favorable light. Social desirability bias could also have influenced the responses, particularly for sensitive questions related to habits such as smoking and alcohol consumption. Furthermore, the cross-sectional design restricts the ability to establish causal relationships between the sociodemographic factors and the levels of stress and sleep deprivation observed. Future studies employing longitudinal designs could provide more insights into these relationships over time.

## Conclusions

The distribution of the study participants according to their comorbidity status reveals that among the 429 drivers, hypertension, diabetes mellitus, and cardiovascular disease were the common conditions reported. Those drivers who were degree holders took less than three breaks in duty time, slept more than six hours at night, had a habit of smoking and drinking alcohol, experienced very poor sleep according to the ESS, and needed medical attention. Those drivers who were Muslims, degree holders, smokers, alcohol consumers, drivers with very poor sleep, and those who had taken more than six days of casual leaves in the past six months experienced high stress according to the PSS-10 and needed medical attention. While the study identified significant associations between stress, sleep deprivation, and factors such as smoking and alcohol consumption, it is important to consider the potential role of unmeasured confounding variables. Factors such as pre-existing health conditions, mental health status, and environmental influences (e.g., work environment) may also contribute to the observed outcomes and should be accounted for in future research. Regular scheduled check-ups, together with efforts to reduce the lifestyle and occupational risk factors, should be implemented for government bus drivers. Special attention should be given to achieving proper ergonomics, which can help decrease the occurrence of serious noncommunicable illnesses and their associated sickness and death rates. It is important to prioritize health education for drivers, focusing on taking regular breaks, avoiding risk factors, and promptly seeking medical attention when needed.
